# Post‐discharge medicines management: the experiences, perceptions and roles of older people and their family carers

**DOI:** 10.1111/hex.13145

**Published:** 2020-10-16

**Authors:** Justine Tomlinson, Jonathan Silcock, Heather Smith, Kate Karban, Beth Fylan

**Affiliations:** ^1^ Medicine Optimisation Research Group School of Pharmacy and Medical Sciences University of Bradford Bradford UK; ^2^ Medicine Management and Pharmacy Services Leeds Teaching Hospitals NHS Trust Leeds UK; ^3^ Faculty of Life Sciences University of Bradford Bradford UK; ^4^ Bradford Institute for Health Research Bradford Teaching Hospitals NHS Foundation Trust Bradford UK

**Keywords:** medication management, medication safety, older people, patient safety, qualitative interviews, transitions of care

## Abstract

**Background:**

Multiple changes are made to older patients’ medicines during hospital admission, which can sometimes cause confusion and anxiety. This results in problems with post‐discharge medicines management, for example medicines taken incorrectly, which can lead to harm, hospital readmission and reduced quality of life.

**Aim:**

To explore the experiences of older patients and their family carers as they enacted post‐discharge medicines management.

**Design:**

Semi‐structured interviews took place in participants’ homes, approximately two weeks after hospital discharge. Data analysis used the Framework method.

**Setting and participants:**

Recruitment took place during admission to one of two large teaching hospitals in North England. Twenty‐seven participants aged 75 plus who lived with long‐term conditions and polypharmacy, and nine family carers, were interviewed.

**Findings:**

Three core themes emerged: impact of the transition, safety strategies and medicines management role. Conversations between participants and health‐care professionals about medicines changes often lacked detail, which disrupted some participants’ knowledge and medicines management capabilities. Participants used multiple strategies to support post‐discharge medicines management, such as creating administration checklists, seeking advice or supporting primary care through prompts to ensure medicines were supplied on time. The level to which they engaged with these activities varied.

**Discussion and conclusion:**

Participants experienced gaps in their post‐discharge medicines management, which they had to bridge through implementing their own strategies or by enlisting support from others. Areas for improvement were identified, mainly through better communication about medicines changes and wider involvement of patients and family carers in their medicines‐related care during the hospital‐to‐home transition.

## 1 INTRODUCTION

Reducing avoidable medicines‐related harm is a global health challenge, and the World Health Organization has called for its reduction to be a priority.^1^ Certain clinical situations are more likely to result in problems with medicines. Transitions in care, where patients are handed over from one clinician to another, for example at hospital discharge, are high risk.^2^ It is estimated that up to 70% of patients experience unintended changes to their medicines at hospital discharge.^3^ These medication errors can result in significant harm for patients and a subsequent cost to the health‐care service.^4^, ^5^, ^6^ Older patients are particularly at risk because they often take multiple medicines for a variety of conditions, sometimes involving different care providers.^7^ Approximately 37% of older people over the age of 65 years are considered to be at risk of medicines‐related harm in the eight weeks following hospital discharge.^8^

Previously, research has sought to identify the types of medicines‐related problems and harm that older patients experience in the post‐discharge phase.^9^, ^10^ Problems obtaining medicines supply, with taking medicines and the effects of poor communication and co‐ordination are common.^6^, ^11^, ^12^ Limited research has been conducted to elucidate older people's lived experience of post‐discharge medicines management, which may help identify gaps in current service provision. Those that have, found that most older patients were confused or anxious about their post‐discharge medicines and were often not involved in medicines‐related decisions.^6^, ^12^, ^13^, ^14^

Optimal medicines management (the actions, processes and behaviours that determine how medicines are used) promotes medicines safety and is thus an important component of effective post‐discharge care.^15^, ^16^ Previous research has demonstrated that interventions provided by health‐care professionals (with or without patient interaction), such as the reconciliation of pre‐ and post‐discharge medicines regimens, may be effective in reducing negative outcomes such as hospital readmission.^9^, ^17^, ^18^, ^19^ Furthermore, cardiology patients and patients within primary care have been shown to perform activities that contribute to post‐discharge medicines management ^20^ and to keep themselves safe.^21^ It is currently unknown, however, what strategies, if any, older patients and their family carers use themselves to support medicines management.

This study aimed to address this gap and explore the experiences of older patients and their family carers as they enacted post‐discharge medicines management, focusing on identifying what helps and hinders them. Exploring these factors will help identify the current medicines management needs and priorities for older people following hospital discharge, which has implications for practice.

## 2 METHODS

### 2.1 Study design

This was a descriptive qualitative study that used semi‐structured interviews to ascertain the opinions, feelings and perspectives of participants. Ethical approval for this study was granted by the Yorkshire and Humber, Bradford Leeds Local Research Ethics Committee, on 3 July 2018, reference 18/YH/0233.

### 2.2 Patient and public involvement in this study

Four older patients and family carers were involved in the conception and development of this study. Their involvement has been fully documented elsewhere.^22^ Briefly, the group co‐designed participant documentation, data collection tools and wording of the interview topic guide with the researcher (JT). They also considered the impact that the research could have for participants, such as any burden associated with in‐depth interviews. We particularly valued their interpretations of transcript excerpts, which prompted new lines of analysis.

### 2.3 Setting and participants

Participants were recruited during their admission to the older people's wards at two large teaching hospitals in the North of England. Patients were eligible to take part if they: were aged 75 years or over; used five or more medicines; lived with long‐term conditions (frailty and type 2 diabetes mellitus were used as exemplar conditions in this study); and had a medicines change during their admission to hospital. Family carers of the participants who helped them with their medicines at home were also invited to take part. People who could not communicate verbally, lacked capacity or those who were discharged to long‐term care facilities were excluded as these were not our population of interest at this time. This study took place between August 2018 and November 2019.

A maximum variation sampling technique ^23^ was used to ensure participants with a range of health‐care support needs and characteristics (for example those living independently to those requiring local authority support, living in sheltered housing to those isolated) were recruited.

### 2.4 Data collection

Older patients who met the inclusion criteria were approached by the researcher during their hospital stay to provide the study information leaflet, answer questions and obtain written informed consent. If they consented to take part, they were contacted approximately two weeks after hospital discharge to reaffirm consent. The researcher visited participants in their home and conducted the semi‐structured interview using a topic guide. The guide was developed from clinical experience of the patient/medicine pathway, literature about patient experiences, and information gathered from practitioner and patient stakeholder conversations. Questions with associated probes and prompts explored topics including medication‐related interactions with health‐care professionals or other organizations, self‐care strategies and examples of post‐discharge medication‐related problems (see Data S1). Participants were also asked about anything that enhanced their experience of medication management since hospital discharge. The interviews began with a story‐telling element, allowing the participant to offer an uninterrupted, rich narrative around their experiences with medicines since hospital discharge.^24^ Interviews were audio‐recorded and transcribed verbatim.

### 2.5 Data analysis

Inductive thematic analysis is a method that is widely used to identify, interpret and report themes in qualitative data.^25^ The Framework method ^26^ differs from traditional thematic analysis as it involves the creation of an analytical framework that is applied and used to chart data into a matrix. This allows the data to be compared across and within individual participants. This ensures the analytical process is much more systematic, auditable and comprehensive, as all participants are considered, not just dominant ones.^27^ The analysis of our data followed the Framework method as outlined by Ritchie et al.^27^ Interviews were coded by JT and managed in groups of seven to make concurrent data collection and analysis manageable. BF independently coded a quarter of all interviews and held frequent discussions with JT to agree thematic development. This also allowed for careful consideration of data saturation. Codes obtained from the first seven interviews were collated and aggregated inductively into an analytic framework to guide the creation of the matrices. We decided against using a deductive approach, where a priori coding frameworks are applied to the interview data, so that all important factors within the interviews were considered and none overlooked.^28^ This analytic plan was reviewed by all authors and adapted to ensure that all codes could fit, resulting in the creation of six matrices, recorded using NVivo 11 software. Interview transcripts for each participant were re‐read, and key issues pertaining to that theme of the matrix were summarized. Any ideas or experiences within the transcripts that would not fit within the matrix were discussed between study authors, and further changes were made to the analytic plan as appropriate. This process continued until data saturation occurred, with no new ideas or themes emerging. The matrices were then individually interrogated to identify the key elements within each summary. The detected elements were iteratively sorted into categories. Higher level categories became themes, and lower level categories became subthemes. The thematic analysis was finally refined through discussion with all the authors.

## 3 FINDINGS

During their hospital admission, 42 patients consented to take part in this interview study. Unfortunately, a proportion of these participants were readmitted to hospital (n = 2), became too ill (n = 4), lost interest (n = 6) or subsequently died (n = 3) before an interview could take place. In total, 27 older participants (21 female; mean age 84 years), along with nine family carers, were interviewed, before data saturation occurred with no new codes arising from the transcripts. The majority of participants were White British (n = 26) with one participant being of Afro‐Caribbean heritage. Participants requested that their family carer be interviewed at the same time as them; therefore, eight interviews (one participant involved two family carers) were conducted with patient‐carer dyads.

All participants had at least one medication change or recommendation made about their medicines (mean 4.6 changes). Some returned home with packages of support provided by the local authority (n = 18) to help them recuperate mobility or for homecare purposes (see Table 1 for full participant characteristics).

**Table 1:** Characteristics of participants (n = 27)

Participant characteristics	N (%)
Gender	
Female	21 (77%)
Male	6 (23%)
Age	
≥75 ‐ <85	16 (59%)
≥ 85 ‐ <95	11 (41%)
Mean	84 years
Length of inpatient stay^a^	
0 – <5 days	4 (15%)
≥5 ‐ < 10 days	10 (38%)
≥10 ‐ <15 days	5 (19%)
≥15 ‐ <20 days	3 (12%)
≥ 20 days	4 (15%)
Mean	11 days
Number of medicines changes	
0 – <5	18 (66%)
≥5 ‐ < 10	6 (22%)
≥10	3 (11%)
Mean	4.6 changes
Lives with	
Alone	19 (70%)
Spouse	7 (26%)
Other	1 (4%)
Lives in	
House	18 (66%)
Flat	4 (15%)
Sheltered accommodation	5 (19%)
Who helps with medicines	
No one	6 (22%)
Family	17 (63%)
Adult social services	3 (11%)
Combination of family and social services	1 (4%)

^a^ Length of stay was unknown for one participant.

### 3.1 Framework analysis

**Figure 1:**
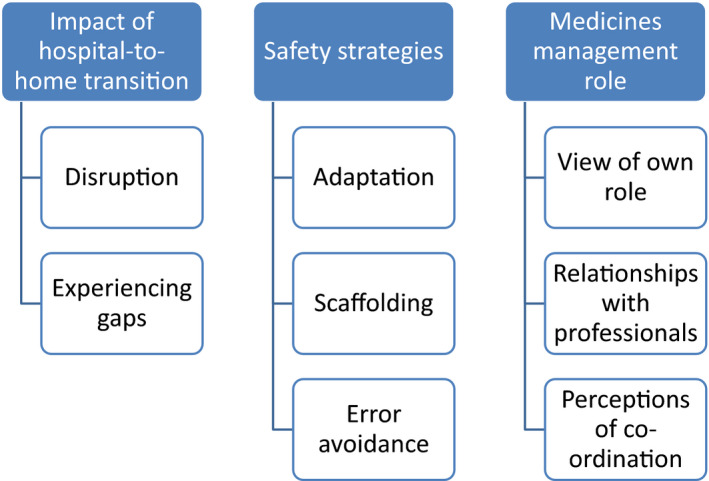
Framework analysis core themes and subthemes

Three thematic areas with eight subthemes (see Figure 1) were identified. The core themes were as follows: impact of the transition, safety strategies and medicines management role. Participants described how the nature of their hospital admission, and subsequent return home with changes to established medicines regimens, disrupted their medicines management. To mitigate issues, participants performed various activities that supported medication safety. The level to which participants performed these activities appeared to depend on their relationship with their medicines, their health‐care professionals and their perceptions of their post‐discharge care. Each theme will be presented in turn, supported by verbatim quotes from participants, using pseudonyms to maintain confidentiality.

## 4 THE IMPACT OF THE HOSPITAL‐TO‐HOME TRANSITION

The first theme includes two subthemes: disruptions and experiencing gaps. Participants described how their transition from the hospital disrupted their medicines‐related knowledge, routines and capabilities. There were gaps, or structural faults in the system, as there was no service or process to help participants re‐orientate or cope with these disruptions.

### 4.1 Disruptions

Following discharge from hospital, participants were unsure which, if any, of their medicines had been changed:

'I think some of them have changed but I’m not sure which…'. [Nancy, 82]

'Yes, they changed one or two, because I was on atenolol when I went in and they stopped that but when I came out they didn’t say'. [Charles, 82]

Where participants had managed their own medicines before admission, multiple regimen changes disrupted the systems that had worked for them beforehand and some found themselves struggling with stock management or to administer their doses. Elizabeth (87) reported how she knew all her medicines before admission; however, by the time of hospital discharge she no longer knew what she was taking, when or why. This greatly impacted on her medicines‐taking abilities and confidence when she returned home:

'… we’d got all these boxes I said, “I can’t cope with that,” you know because I just didn’t know what they were'.

Fluctuating health, post‐discharge deterioration and prolonged recovery also affected participants’ medicines management capabilities. In the early post‐discharge phase, they often did not feel well, forgot to take their medicines or struggled to access their doses due to lower levels of mobility:

''When I got home I were thinking to myself I think they’ve let me out too early; I’m not ready, you know, because I didn’t feel… I didn’t feel as if I could cope, you know, with everything and I was forgetting my tablets…'. [William, 79]

### 4.2 Experiencing gaps

A lack of quality conversations during hospital admission was noticed by most participants. They described how they had not known what was happening, perceived clinicians to be withholding information or felt that medicines had been changed without permission:

'I accept when they tell me, what I don’t like is doing it behind my back and thinking that an old codger of 80, he won’t remember what his tablets were; I do'. [Robert, 80]

'Nobody explained anything. I didn’t even know they took me off the rivaroxaban'. [Winifred, 78]

Conversations at discharge were also limited. For example, Robert (80) had his new medicines sent out to him via a taxi, with no one speaking to him about this treatment. Others, like James (79), were not provided with any explanation despite receiving their medicines on the ward; '*I was just given the bag of medication and that [discharge letter]'*. Family carers reported similar experiences:

'when we got there they'd just gone for the tablets, so it's just like yeah, there's your tablets, off you go'. [Elaine's (96) daughter]

This left participants and their family carers with unanswered questions about their conditions, why changes had been made, what they had to do once they had returned home or what side effects to anticipate:

'…but you still don’t know why they took her in twice and why, you know, she’s had the stopping of the cholesterol and the acid tablet, you used to take two and now they’ve changed that just to one'. [Betty’s (85) daughter]

'I did say to someone “Has my medicines changed?” and they said “We don’t know” but they have changed'. [Hazel, 91]

Following discharge, participants encountered issues with: delayed supplies, ordering new items, oversupply of things no longer needed and incorrect medicines within their multicompartment compliance aids. Problems with follow‐up and care co‐ordination within the community were also frequent; for example, Shirley (81) described being unclear who her follow‐up appointments were with, or why she needed to attend; Joan (78) was concerned her blood pressure had not been reviewed since the hospital had stopped her blood pressure medication; and in Eleanora's (83) case, there was a failure to restart homecare services.

## 5 SAFETY STRATEGIES

To mitigate these disruptions, participants proactively developed techniques and activities to support post‐discharge medicines management. These strategies involved adapting pre‐admission medicines administration routines, reaching in to proactively support the health‐care system and being vigilant.

### 5.1 Adaptation

Changes to regular routine, or home environment after hospital discharge, allowed participants to take control and manage their own medicines. Routine appeared important for many, and participants described how they developed new post‐discharge regimens in relation to their meals or personal daily milestones:

'I’ve started to take my medication different now, different times [...] so I get up about half past six in a morning and make myself a cup of coffee, and then just wait a bit and take another couple of tablets and then I take another one that leaves me two after I’ve had my breakfast'. [Betty, 85]

Furthermore, some participants changed the way they performed their often complex medicines‐taking behaviours. Doris (88), for example, preferred to sit at her armchair and work through her discharge paperwork, popping the required tablets into a pot before taking them. Enid (81) decided to set her daily medicines out on a circular tray, depicting the hours of the day when she preferred to take them. Joan (78) used to keep all her medicines in her bedroom, but since discharge, she preferred to keep them all together by her armchair:

'I keep them in the magazine rack, they’re in a sandwich box in magazine rack, so they’re in order, [I] go round sort of thing…'. [Joan, 78]

### 5.2 Scaffolding

Scaffolding activities involved participants, family carers and in some instances clinicians, who proactively provided temporary support to the participant or the health‐care system.^29^ For example, Betty's (85) and Elaine's (96) daughters acted as conduits for information and took the discharge paperwork to the primary care practitioner and/or community pharmacist as soon as possible after discharge. Alice (92) alerted the community pharmacist that she had been discharged so that the medication supply could be restarted. Hazel (91) and Dorothy (82) went one step further and took their discharge medication supply into the pharmacy to show them what their new medicines were:

'They (hospital) gave me some more [medication] to take which I take to the chemist and let them know'. [Dorothy, 82]

Temporary or impromptu strategies were also used in the early post‐discharge period to support safe administration of medicines, whilst participants were recuperating. Marie's (81) daughter annotated the calendar when doses were due to change, and Mary's (81) husband produced checklists to show what medicines should be taken and when. James’ (79) daughter helped him to rationalize his stock of medicines, and Elizabeth's (87) daughter set out her medicines in individual pots, every day, until she was used to the new regimen.

### 5.3 Error avoidance

Participants and their family carers carried out activities that aimed to prevent medication errors. They sought information and took time to study their new medicines in order to grow in confidence with them. They did this by reading medicines information leaflets, using the Internet or asking their clinicians for further information:

'…because on the lid it says what you’re taking doesn’t it, and when you’re taking them? So, we did read that to make sure that they were the ones that I should be taking'. [Elizabeth, 87]

Participants were also able to anticipate potential gaps in onward care from their previous experiences of being in hospital. If they had concerns about certain elements of post‐discharge care, they then defended against them by asking specific questions to the care team. This enabled them to better prepare themselves for post‐discharge medicines management:

'Well yeah, ‘cause it’s belt and braces, I needed to make sure that they’d got the right you know…[…]… That’s why I rang them ‘cause I thought if there’s going to be any delay I’ll just go and pick it up myself'. [Patricia’s (85) daughter]

'…because many a time I’ve read up where people have been given the wrong medication, so to be safe, for my peace of mind, nobody else’s, I like to question each tablet'. [Marie, 81]

Close stock control also ensured appropriate quantities of medicines were available to mitigate against supply issues, such as late delivery by the community pharmacy. Mary's (81) husband made sure to have at least two months of each medicine in stock. Robert (80) and James (79) separated out their stopped medicines from the current supplies, intending to return them to the community pharmacy. Others, such as Marie (81) and Joan (78), had too much stock at home due to oversupply and took active steps to reduce or stop this. Hazel (91) proactively returned her old medicines to the community pharmacy immediately after changes had been made.

## 6 MEDICINES MANAGEMENT ROLE

How participants perceived their role in medicines management determined how they enacted safety strategies; how they enacted their role was determined by the view of the importance of their medicines, the relationship with the primary care provider, and the co‐ordination of their care.

### 6.1 The view of their own role

Participants appeared to assume a range of roles within their post‐discharge medicines management. Some were actively engaged in medicines management and wished to be fully informed and involved in decisions about their medicines. They described how they were not afraid to tell someone if they were unhappy about issues related to their medicines and felt empowered to ask questions of health‐care professionals:

'If I don’t like something and I don’t see… I want to know why not. Like I say, it’s the only way you find out stuff is to ask. If you don’t ask, you don’t know'. [Marie, 81]

'Like I asked about the leg; this is my leg not yours, so I don’t care what you think about that, if I’m not happy about it I will tell you and I will ask you to see your superior'. [James, 79]

Other participants, such as Enid (81) and Ruth (90), had lived with their condition(s) for a long time, which prompted them to learn about what their medicines did and how they worked. They often prioritized certain medicines that were important to them (eg. for heart and blood) and therefore could describe fully any associated medicines changes. For these participants, medicines management required effort and significant daily work:

'But it’s like a job really, if you know what I mean? I know it’s silly but it’s like having a job, oh I’ve got to break off and do this [my medicines], you know?' [Enid, 81]

Despite having to repeatedly scaffold the system to ensure she received her medicines on time, Alice (92) firmly believed that she should not have sole responsibility for her medicines; '*I mean I have to do it [meds management] all myself you see? And it's not right at my age'*. Furthermore, a few participants simply could not manage their medicines and relied on others, such as formal carers, to administer them. This was often due to their feelings of deteriorating memory or reduced capability after discharge, such as Margaret (82): '*No I leave it [medicines] for the carers…[…]… It's something I won't be doing'* and Elsie (84): '*No, I couldn't now, no, my brain's not there; half of it is missing'*.

Other participants administered their own medicines but did not appear concerned about the medicines that they took. Hazel (91) had adopted this attitude following her numerous hospital admissions and subsequent changes to her medicines; '*…every time I go in hospital some doctor or other changes some tablets. I think because they change every time you go, just take them, so that's all I do'*.

### 6.2 Relationships with health‐care professionals

Negative relationships with health‐care professionals led to a lack of trust in clinicians to perform their role, or left the participant feeling that they could not call on them for advice. Some of the participants had very strained relationships with their primary care provider, and this prompted a lack of engagement with their clinicians and sometimes with their medicines:

*'No, no, I’ve never seen him (the General Practitioner (GP)). I were thinking about that last night whether to get in touch with them again or tell them at clinic on Monday and I thought well the best will be to go to clinic because I can spend three‐quarters of an hour and not get through (on the telephone)' [William, 79]*.

Community pharmacists were sometimes seen as the ‘supplier’ or ‘dispenser’ of medicines. Some participants, such as Harry's (90) daughter, Patricia's (85) daughter and Robert (80), explained that they would not consider asking their pharmacist for advice or information beyond this supply function. Those participants who described more long‐standing, positive relationships trusted their pharmacist to explain things fully, valued the service that they received and felt well looked after. Participants therefore often took steps, such as contacting the pharmacy after discharge, to ensure they were kept up to date with discharge information.

### 6.3 Perceptions of care co‐ordination

Many participants believed that information about their admission was transferred between the hospital, community pharmacy and their primary care provider. Whilst they assumed this was the case, they did not know by which processes this might occur. Mary (81), for example, firmly believed that follow‐up plans were in place, and Joan (78) believed that important day‐to‐day information was passed between key individuals within primary care (for example her district nurse was passing information to the GP), although they had no evidence that this was the case:

'We are expecting to attend a follow up… I know our GP will have received a copy of this and that’s it isn’t it. Just have to wait and see now'. [Mary, 81]

'…well they (GP) just go by what district nurses tell them about this. And I suppose they know and the home care as well, everything is written down in that book (nurse’s notes kept in the home)…'. [Joan, 78]

Other participants perceived their care as disorganized, bordering on chaotic. Some felt that they had to take charge and co‐ordinate care by themselves, especially since they did not trust the system to do what it is supposed to. They were frustrated at needing to continually follow things up and described the burden that this added. Some perceived there to be poor communication between sectors, which led them to believe that they must act as conduits:

'I mean it is just that annoying when you have to keep ringing and thinking why but it’s not the first time I’ve had to go over the medication in [the] doctor’s surgery'. [Elaine’s (96) daughter]

'I said, “They told me it was sent late, say mid‐afternoon. Now they're saying they haven't received it.” …[…]…So why? Who is telling stories? It took two days to get a prescription'. [Betty’s (85) husband]

## 7 DISCUSSION

This study aimed to explore the perceptions of older people and their carers, about their post‐discharge medicines management. We found three main themes and eight subthemes describing how a hospital stay, followed by transition home with changed medication, was experienced as a disruption to knowledge, routine and capability. This affected participants’ medicines management, and various strategies were used to help support medicines use.

The findings from our analysis add to the body of literature about post‐discharge medicines management, which spans the last ten years.^6^, ^9^, ^12^, ^13^, ^14^, ^16^ Deficiencies in medicines conversations and information provision at discharge significantly affected participants’ abilities to manage their medicines. Knight et al similarly concluded that most participants in their study reported little or no provision of information about changed medicines, which left them feeling disappointed or confused about their discharge medicines.^6^ Obtaining an overview of medicines changes has previously been found problematic for older people living with frailty in Denmark.^13^ Conversations detailing medication changes are valuable, especially to older patients who are likely to have multiple changes to their regimens throughout their inpatient admission.^30^ This ensures that they understand what has been changed and what they need to do differently. This present study, however, has shown that participants felt these conversations were often lacking detail, did not answer their question or simply did not happen at all.

To work around subsequent medicines‐related problems or gaps in care, participants described using various strategies and techniques, which helped them promote the safe use of post‐discharge medicines. Similar strategies have been found in medicines management work performed by cardiology patients, where they anticipated discrepancies and mitigated their occurrence by facilitating communication between care settings.^31^ Furthermore, research with heart failure patients found similar experiences which identified the props, or strategies participants used to overcome gaps in the system.^32^ Experienced carers within our study explained how they used pre‐prepared lists of questions to ensure that they found out the appropriate details that they would need to support onward medicines management. This foresight may be beneficial since questions often arise after the transition home, when patients and carers resume their on‐going care activities and they are unsure where to seek answers.^31^ In this study, most participants and their carers attempted to seek information for their unanswered questions at a later stage, following discharge. This was challenging as care had been handed over to a different clinician in a different setting. Research should therefore be conducted to identify how to better prepare older patients and their family carers for post‐discharge medicines management.

Interestingly, previous work has emphasized that older people living with frailty do want to be involved in their care; however, it has remained unclear how and to what extent.^33^ This study has demonstrated a range of activities that older patients can and do engage with. Current patient safety literature advocates the engagement of patients as partners in their care in order to overcome threats to safety and to improve patient‐centred outcomes.^29^, ^34^, ^35^ Furthermore, low levels of patient participation are associated with an 8% to 21% higher health‐care cost.^36^ Hence, health‐care professionals should work together with patients to encourage shared decision making. Previous literature, however, has identified that patients over 65 years preferred less engagement in their care whilst in hospital ^37^, ^38^, ^39^ and less information about their medicines.^40^ Whilst this may be true for some older patients, our findings demonstrate a range of preferences for engagement, and therefore, the level of need and desire should be considered for each individual patient. Furthermore, if medicines conversations are not held with patients after discharge, medicines may be left without continual review for a long time. This also limits the opportunities for the patient to be involved in the shared decisionmaking process that should occur regularly along with medication reviews. Belcher et al identified three categories of medicines decision making in the older patient: those who do not want to participate; those who cannot; and those who can and should participate.^41^ Parallels can be drawn to the current study, where a similar range of roles within medicines management behaviours were found. Our analysis, however, demonstrated that the roles were not static. Some participants were able to escalate their participation to meet the additional challenges the disruption to their routines presented, for example by implementing safety strategies. Therefore, it must not be assumed that patients will always perform one role, and similarly, any interaction with the system must identify the current role and the desire to move on.^42^

Willingness to participate is also dependent on the task at hand ^41^ and trust in the health‐care professional; ^43^ therefore, careful exploration of what post‐discharge medicines management involves for the patient should be performed as part of the hospital discharge process. A study showed that 90% of patients (n = 100) were willing to trial deprescribing if their doctor thought it appropriate.^43^ Eassey et al argue that it is the responsibility of health‐care professionals to assess their patient's preference for this level of engagement; however, there are limited validated strategies available to do this.^44^ Flink et al further demonstrated that health‐care professionals are key to encouraging the engagement of patients and family carers, most often through supportive conversations.^38^ Within the present study, conversations about medicines, beyond ascertaining a medicines history at admission, were not perceived to have occurred.

Involving patients throughout their journey in order to resolve any knowledge and skill deficits appears to be one way to ensure that patients, and their family carers, have the tools to enact medicines management. Other interventions, such as patient education and services to reconcile old and new medicines, have been proposed to better help patients prepare for discharge and to support successful hospital‐to‐home transitions.^17^, ^18^, ^19^ A recent systematic review has further illustrated the importance of communication and engagement with patients across the transition, rather than just at discharge, with components describing self‐management activities being most effective.^45^ Whilst it is clear that patients and their carers are able to do certain tasks to promote medicines safety, it is unclear what discrete activities contribute to the self‐management of medicines. Furthermore, there is a lack of evidence as to what behaviours contribute to successful self‐management and how clinicians can coach patients, at the various levels of engagement, towards this. A useful model for further exploration in this context is that of ‘mindful organization’.^46^ Patients can contribute to medicines safety through: applying knowledge about medicines risks, communicating with health‐care professionals, using artefacts, such as medicines leaflets and labels, and recognizing levels of trust.

### 7.1 Implications for practice

This study demonstrates that opportunities to engage with older patients as a resource for successful post‐discharge medicines management are missed, for example, through appropriate conversations throughout the hospital stay (including discharge) and shared decision making. In order to promote wider involvement of older people in their medicines care, established services (such as structured medication reviews or advice about new medicines) or support for patients should be visible, accessible and suitable. For example, current services within England that support post‐discharge medicines management often rely on the patient to proactively visit their community pharmacy or on a pharmacy team to identify which patients require support and to contact them via telephone as part of their already busy workload.^47^

Some progress has been made however, with a new UK discharge service announced by the Department of Health and Social Care.^48^ This appears to focus on the transfer of information from the hospital to community pharmacy, similar to services already available in some places.^49^, ^50^ Whilst it is unknown what is available within this service, it currently does not appear to integrate the patient within it. Given the lack of engagement and involvement highlighted by this study, it would be beneficial to rethink future services and ensure they are truly person‐centred.

### 7.2 Methodological considerations

This study involved a moderate sample size, which allowed the deep exploration of rich and varied experiences. The sample was limited in its ethnic diversity and does not represent the wider population of the UK. It is therefore unclear whether the findings are transferrable to other patient groups and to the population as a whole. Whilst made up of predominantly White British participants, we achieved the recruitment of a varied sample with regard to medicines management needs.

The first author conducted the framework analysis, supervised by BF who read and independently coded 25% of transcripts. All authors were involved in the development and refinement of the final thematic areas. Reflexivity is presented so that the lens through which the analysis was observed is clear. JT is an older people's pharmacist, who may have influenced interview conduct and subsequent analysis; however, the Framework method^26^, ^27^ ensured that we remained true to the content of the interviews. Interviews were conducted two weeks after hospital discharge, which may have affected participant's recall of events.

## 8 CONCLUSION

This study has provided an in‐depth exploration of older patient's experiences of post‐discharge medicines management. Participants faced significant disruptions to their medicines knowledge and capabilities, which impacted on their management abilities once home. They also perceived gaps in their onward care, which some participants were able to mitigate against by developing their own strategies to support medication safety. Opportunities exist to involve and engage older patients living with frailty and, where appropriate, their family carers, during their hospital stay and in the post‐discharge period, to ensure effective medicines management.

## CONFLICT OF INTEREST

The authors declare that they have no conflict of interest.

## AUTHOR CONTRIBUTIONS

JT with supervision from all authors performed material preparation and data collection. JT and BF, with JS, HS and KK providing comments conducted data analysis. JT wrote the first draft of the manuscript. All authors commented on previous versions of the manuscript, read and approved the final manuscript, and contributed to the study conception and design.

## Supporting information

Supplementary Material

## Data Availability

The data that support the findings of this study are available on request from the corresponding author. The data are not publicly available due to privacy or ethical restrictions.

## References

[1] Donaldson LJ , Kelley ET , Dhingra‐Kumar N , Kieny M‐P , Sheikh A . Medication without harm: WHO's third global patient safety challenge. The Lancet. 2017;389(10080):1680‐1681. 10.1016/S0140-6736(17)31047-4 28463129

[2] World Health Organisation . WHO Global Patient Safety Challenge: Medication Without Harm. 2017 https://www.who.int/patientsafety/medication-safety/medication-without-harm-brochure/en/. [Accessed 14th January 2019]

[3] Royal Pharmaceutical Society . Keeping patients safe when they transfer between care providers – getting the medicines right. London: Royal Pharmaceutical Society. 2012 http://www.rpharms.com/current-campaigns-pdfs/rps-transfer-of-care-final-report.pdf. [Accessed 30th July 2016]

[4] Elliott RA , Camacho E , Campbell F , et al. Prevalence and economic burden of medication errors in the NHS in England: Rapid evidence synthesis and economic analysis of the prevalence and burden of medication error in the UK. Policy Research Unit in Economic Evaluation of Health & Care Interventions (EEPRU). 2018 http://www.eepru.org.uk/wp-content/uploads/2018/02/eepru-report-medication-error-feb-2018.pdf. [Accessed 14th January 2019].

[5] Forster AJ , Murff HJ , Peterson JF , Gandhi TK , Bates DW . Adverse Drug Events Occurring Following Hospital Discharge. J Gen Intern Med. 2005;20(4):317‐323. 15857487 10.1111/j.1525-1497.2005.30390.x PMC1490089

[6] Knight DA , Thompson D , Mathie E , Dickinson A . ‘Seamless care? Just a list would have helped!’ Older people and their carer’s experiences of support with medication on discharge home from hospital. Health Expect. 2013;16(3):277‐291. 21838834 10.1111/j.1369-7625.2011.00714.x PMC5060666

[7] Oliver D , Foot C , Humphries R . Making our health and care systems fit for an ageing population. London: The Kings Fund 2014 https://www.kingsfund.org.uk/sites/files/kf/field/field_publication_file/making-health-care-systems-fit-ageing-population-oliver-foot-humphries-mar14.pdf. [Accessed 27th June 2017].

[8] Parekh N , Ali K , Stevenson JM , et al. Incidence and cost of medication harm in older adults following hospital discharge: a multicentre prospective study in the UK. Br J Clin Pharmacol. 2018;84(8):1789‐1797. 29790202 10.1111/bcp.13613 PMC6046489

[9] Garcia‐Caballos M , Ramos‐Diaz F , Jimenez‐Moleon JJ , Bueno‐Cavanillas A . Drug‐related problems in older people after hospital discharge and interventions to reduce them. Age Ageing. 2010;39(4):430‐438. 20497947 10.1093/ageing/afq045

[10] Coleman EA , Smith JD , Raha D , Min S . Posthospital medication discrepancies: Prevalence and contributing factors. Arch Intern Med. 2005;165(16):1842‐1847. 16157827 10.1001/archinte.165.16.1842

[11] Nicosia FM , Spar MJ , Stebbins M , et al. What is a medication‐related problem? A qualitative study of older adults and primary care clinicians. J Gen Intern Med. 2020;35(3):724‐731. 31677102 10.1007/s11606-019-05463-z PMC7080912

[12] Parekh N , Gahagan B , Ward L , Ali K . ‘They must help if the doctor gives them to you’: a qualitative study of the older person’s lived experience of medication‐related problems. Age Ageing. 2019;48(1):147‐151. 30165466 10.1093/ageing/afy142

[13] Andreasen J , Lund H , Aadahl M , Sørensen EE . The experience of daily life of acutely admitted frail elderly patients one week after discharge from the hospital. Int J Qualitative Studies Health Well‐being. 2015:10. 10.3402/qhw.v10.27370 PMC4452652 26037333

[14] Ensing H , Koster ES , Berkel PI , Dooren AA , Bouvy ML . Problems with continuity of care identified by community pharmacists post‐discharge. J Clin Pharm Ther. 2017;42(2):170‐177. 27943349 10.1111/jcpt.12488

[15] Kripalani S , Jackson AT , Schnipper JL , Coleman EA . Promoting effective transitions of care at hospital discharge: A review of key issues for hospitalists. J Hosp Med. 2007;2(5):314‐323. 17935242 10.1002/jhm.228

[16] Care Quality Commission . Managing patients' medicines after discharge from hospital: National study. 2009 https://webarchive.nationalarchives.gov.uk/20101122140156/http://www.cqc.org.uk/_db/_documents/Managing_patients_medicines_after_discharge_from_hospital.pdf. [Accessed 27th June 2017].

[17] Laugaland K , Aase K , Barach P . Interventions to improve patient safety in transitional care–a review of the evidence. Work. 2012;41(Suppl 1):2915‐2924. 22317162 10.3233/WOR-2012-0544-2915

[18] Spinewine A , Claeys C , Foulon V , Chevalier P . Approaches for improving continuity of care in medication management: a systematic review. Int J Qual Health Care. 2013;25(4):403‐417. 23639854 10.1093/intqhc/mzt032

[19] Leppin AL , Gionfriddo MR , Kessler M , et al. Preventing 30‐day hospital readmissions: A systematic review and meta‐analysis of randomized trials. JAMA Intern Med. 2014;174(7):1095‐1107. 24820131 10.1001/jamainternmed.2014.1608 PMC4249925

[20] Fylan B , Armitage G , Naylor D , Blenkinsopp A . A qualitative study of patient involvement in medicines management after hospital discharge: an under‐recognised source of systems resilience. BMJ Quality & Safety. 2018;27(7):539. 10.1136/bmjqs-2017-006813 29146681

[21] Rhodes P , McDonald R , Campbell S , Daker‐White G , Sanders C . Sensemaking and the co‐production of safety: a qualitative study of primary medical care patients. Sociol Health Illn. 2016;38(2):270‐285. 26547907 10.1111/1467-9566.12368

[22] Tomlinson J , Medlinskiene K , Cheong V , Khan S , Fylan B . Patient and public involvement in designing and conducting doctoral research: the whys and the hows. Res Involv Engagem. 2019;5(23): 10.1186/s40900-019-0155-1 PMC6697942 31428458

[23] Davis C , Gallardo HL , Lachlan KL . Sampling In: Straight Talk in Communication Research Methods. Iowa: Kendall Hunt; 2009 171.

[24] Wong C , Hogan DB . Care transitions: Using narratives to assess continuity of care provided to older patients after hospital discharge. Canadian Geriatrics J. 2016;19(3):97‐102. 10.5770/cgj.19.229 PMC5038931 27729948

[25] Braun V , Clarke V . Using thematic analysis in psychology. Qual Res Psychol. 2006;3(2):77‐101.

[26] Gale NK , Heath G , Cameron E , Rashid S , Redwood S . Using the framework method for the analysis of qualitative data in multi‐disciplinary health research. BMC Med Res Methodol. 2013;13(1):117. 24047204 10.1186/1471-2288-13-117 PMC3848812

[27] Ritchie J , Lewis J , McNaughton‐Nicholls C , Ormston R . Qualitative Research Practice: A Guide for Social Science Students and Researchers. London: Sage Publications; 2013.

[28] McGowan LJ , Powell R , French DP . How can use of the Theoretical Domains Framework be optimized in qualitative research? A rapid systematic review. Br J Health Psychol. 2020;25(3):677‐694. 32558289 10.1111/bjhp.12437

[29] O’Hara JK , Aase K , Waring J . Scaffolding our systems? Patients and families ‘reaching in’ as a source of healthcare resilience. BMJ Quality & Safety. 2019;28(1):3. 10.1136/bmjqs-2018-008216 29764929

[30] Harris CM , Sridharan A , Landis R , Howell E , Wright S . What Happens to the Medication Regimens of Older Adults During and After an Acute Hospitalization? J Patient Safety. 2013;9(3):150‐153. 10.1097/PTS.0b013e318286f87d 23965837

[31] Cain CH , Neuwirth E , Bellows J , Zuber C , Green J . Patient experiences of transitioning from hospital to home: An ethnographic quality improvement project. J Hosp Med. 2012;7(5):382‐387. 22378714 10.1002/jhm.1918

[32] Fylan B , Marques I , Ismail H , et al. traps, bridges and props: a mixed‐methods study of resilience in the medicines management system for patients with heart failure at hospital discharge. BMJ Open. 2019;9: 10.1136/bmjopen-2018-023440 PMC6377507 30782879

[33] Ipsos Mori for Age UK . Understanding the lives of older people living with frailty ‐ a qualitative investigation. 2014 https://www.ipsos.com/ipsos-mori/en-uk/understanding-lives-older-people-living-frailty. [Accessed 14th January 2019].

[34] World Health Organisation . Medication Safety in Transitions of Care. 2019 https://apps.who.int/iris/bitstream/handle/10665/325453/WHO-UHC-SDS-2019.9-eng.pdf?ua=1. [Accessed 14th January 2019].

[35] NHS Improvement . The NHS Patient Safety Strategy. 2019 https://improvement.nhs.uk/documents/5472/190708_Patient_Safety_Strategy_for_website_v4.pdf. [Accessed 14th January 2019].

[36] Hibbard JH , Greene J , Overton V . Patients with lower activation associated with higher costs; delivery systems should know their patients’ ‘Scores’. Health Aff. 2013;32(2):216‐222. 10.1377/hlthaff.2012.1064 23381513

[37] Mohsin‐Shaikh S , Garfield S , Dean‐Franklin B . Patient involvement in medication safety in hospital: an exploratory study. Int J Clin Pharm. 2014;36:657‐666. 24777838 10.1007/s11096-014-9951-8 PMC4019827

[38] Flink M , Öhlén G , Hansagi H , Barach P , Olsson M . Beliefs and experiences can influence patient participation in handover between primary and secondary care—a qualitative study of patient perspectives. BMJ Qual Saf. 2012;21:27‐83. 10.1136/bmjqs-2012-001179 PMC3551196 23112289

[39] Levinson W , Kao A , Kuby A , Thisted RA . Not all patients want to participate in decision making. J Gen Intern Med. 2005;20:531‐535. 15987329 10.1111/j.1525-1497.2005.04101.x PMC1490136

[40] Duggan C , Bates I . Medicine information needs of patients: the relationships between information needs, diagnosis and disease. BMJ Quality & Safety. 2008;17:85‐89. 10.1136/qshc.2005.017590 18385399

[41] Belcher VN , Fried TR , Agostini JV , Tinetti M . Views of older adults on patient participation in medication‐related decision making. J Gen Intern Med. 2006;21:298. 16686804 10.1111/j.1525-1497.2006.00329.x PMC1484726

[42] Carmen KL , Dardess P , Maurer M , et al. Patient and family engagement: a framework for understanding the elements and developing interventions and policies. Health Aff. 2013;32(2):223‐231. 10.1377/hlthaff.2012.1133 23381514

[43] Reeve E , Wiese MD , Hendrix I , Roberts MS , Shakib S . People’s attitudes, beliefs, and experiences regarding polypharmacy and willingness to deprescribe. J Am Geriatr Soc. 2013;61:1508‐1514. 24028356 10.1111/jgs.12418

[44] Eassey D , McLachlan AJ , Brien J , Krass I , Smith L . “I have nine specialists. They need to swap notes!” Australian patients’ perspectives of medication‐related problems following discharge from hospital. Health Expect. 2017;20:1114‐1120. 28306185 10.1111/hex.12556 PMC5600251

[45] Tomlinson J , Cheong V , Fylan B , et al. Successful care transitions for older people: a systematic review and meta‐analysis of the effects of interventions that support medication continuity. Age Ageing. 2020;00:1‐12. 10.1093/ageing/afaa002 PMC7331096 32043116

[46] Phipps D , Giles S , Lewis PJ , et al. Mindful organizing in patients’ contributions to primary care medication safety. Health Expect. 2018;21:964‐972. 29654649 10.1111/hex.12689 PMC6250879

[47] Rutter P , Ramsbottom H , Fitzpatrick R . Community pharmacist perceptions of delivering post‐hospital discharge Medicines Use Reviews for elderly patients. Int J Clin Pharm. 2017;39:33‐36. 27905074 10.1007/s11096-016-0400-8

[48] Pharmaceutical Services Negotiating Committee . Discharge Medicines Service. 2020 . https://psnc.org.uk/services-commissioning/essential-services/discharge-medicines-service/. [Accessed 28th May 2020].

[49] Sabir FRN , Tomlinson J , Strickland‐Hodge B , Smith H . Evaluating the Connect with Pharmacy web‐based intervention to reduce hospital readmission for older people. Int J Clin Pharm. 2019;41:1239‐1246. 31392581 10.1007/s11096-019-00887-3 PMC6800861

[50] Nazar H , Brice S , Akhter N , et al. New transfer of care initiative of electronic referral from hospital to community pharmacy in England: a formative service evaluation. BMJ Open. 2016;6(10):1‐9. 10.1136/bmjopen-2016-012532 PMC5073802 27742628

