# Supplementary material for: Post‐discharge medicines management: the experiences, perceptions and roles of older people and their family carers
Source: Health Expect. 2020 Oct 16;23(6):1603–13. doi: 10.1111/hex.13145 (PMC7752204; doi:10.1111/hex.13145)
Supplement: Supplementary file 1 — Supplementary Material [file HEX-23-1603-s001.docx]

**Supplementary material 1 -**

**Topic Guide – Interview: 2 weeks post-discharge**

Participant identifier: Date:

*Introduction:*

My research is interested in what happens to patient’s medicines when they move from hospital to home. I am really keen to hear how you have been getting on with your medicines since your stay in hospital.

I’d like to ask you some questions about your experiences with your medicines. This will take approximately 40 minutes. If you would like a break or want to stop for any reason at all, then please do let me know.

[Check audio and video recorder]

*Story-telling narrative:*

Q Would you like to start by telling me a little bit about your hospital stay and how you got on with your medicines on returning home?

Prompt: What happened next?
 Prompt: What did you think/ feel about that?
 Prompt: How has this affected you?

---------

Q Can you please tell me a bit about the different medicines you take?

Prompt: Were there any changes made whilst you were in hospital?
 Prompt: Were these changes explained to you?

Q What was explained to you about what would happen with your medicines when you returned home?

Prompt: What were you told about your further medicines supply for when you returned home?
 Prompt: How did you find out?
 Prompt: If you hadn’t have been told any information, what would you have done?

Q Is there anything else you would have liked to have been told about your medicines when leaving the hospital?

Prompt: What did you find helpful?

Q Have you experienced any problems with your medicines since returning home?

 Prompt: How you manage the ordering of your medicines?
 Prompt: And how do you get on taking them?
 Prompt: Who helps you with your medicines?

Q Have you spoken to anyone about your medicine changes since you have been in hospital for example your GP or pharmacist?

Prompt: Do you intend to?
 Prompt: Why?

Q How do you make sure you continue to get the right medicines since the changes have been made?

Prompt: How easy or difficult is it to manage your medicines after you have been in hospital?
 Prompt: Do you think there are there any special skills required to do this?

Q How do you make sure that your medicines are looked after?

Prompt: Is there anything that you do routinely that helps you to manage your medicines?
 Prompt: What prevents you from doing this?
 Prompt: Are there any strategies that could help you to overcome this?
 Prompt: Is there any reward or incentive that would encourage you to manage and take your medicines?

Closing statement, thank the participant.

Duration of interview:

Audio number:

**Supplementary material 2 - COREQ checklist**

| **Domain 1: Research team and flexibility** |  | **Location in manuscript (section, pg. number)** |
| --- | --- | --- |
| **Personal characteristics** |  |  |
| 1. Interviewer/facilitator Which author/s conducted the interview or focus group? | JT | Methods (data collection) - 3 |
| 2. Credentials  What were the researcher’s credentials? E.g. PhD, MD | JT is a practicing pharmacist and doctoral researcher. This work forms part of her doctoral studies, with support provided by: BF, an experienced health services researcher; JS, a pharmacist academic and researcher; HS, a consultant pharmacist for older people; and KK, a qualitative researcher and social work academic. | - |
| 3. Occupation  What was their occupation at the time of the study? | Pharmacist – care of the elderly | - |
| 4. Gender Was the researcher male or female? | Female | - |
| 5. Experience and training  What experience or training did the researcher have? | JT has undergone formal training courses in qualitative interview methods (Social Research Association) and Framework analysis (Natcen: Qualitative Data Training). | - |
| **Relationship with participants** |  |  |
| 6. Relationship established  Was a relationship established prior to study commencement? | Yes - JT conducted each aspect of the study including participant recruitment, telephone contact to arrange interviews and conducted all interviews. This helped the development of meaningful researcher-participant relationships, which was perceived to encourage participants to be open in interviews. | - |
| 7. Participant knowledge of the interviewer  What did the participants know about the researcher? e.g. personal goals, reasons for doing the research | Participants were briefed on the purpose of the study during recruitment and via participant information sheet. They understood it was a study for JT’s PhD. | - |
| 8. Interviewer characteristics What characteristics were reported about the interviewer/facilitator? e.g. Bias, assumptions, reasons and interests in the research topic | Participants knew JT was a pharmacist from the information sheet. No other interviewer biases identified. | Discussion (methodological considerations) - 15 |
| **Domain 2: Study design** |  |  |
| **Theoretical framework** |  |  |
| 9. Methodological orientation and Theory  What methodological orientation was stated to underpin the study? e.g. grounded theory, discourse analysis, ethnography, phenomenology, content analysis | Inductive framework analysis | Methods (data analysis) - 4 |
| **Participant selection** |  |  |
| 10. Sampling How were participants selected? e.g. purposive, convenience, consecutive, snowball | A purposive maximum variation sampling technique guided recruitment. | Methods (setting and participants) - 3 |
| 11. Method of approach How were participants approached? e.g. face-to-face, telephone, mail, email | Face to face | Methods (data collection) - 4 |
| 12. Sample size How many participants were in the study? | 27 | Findings - 5 |
| 13. Non-participation How many people refused to participate or dropped out? Reasons? | During their hospital admission, 42 patients consented to take part in this interview study. Unfortunately, 15 of these participants were readmitted to hospital (n=2), became too ill (n=4), lost interest (n=6) or subsequently died (n=3) before an interview could take place. | Findings - 5 |
| **Setting** |  |  |
| 14. Setting of data collection  Where was the data collected? e.g. home, clinic, workplace | Interviews took place in the participant’s home. | Methods (data collection) - 4 |
| 15. Presence of non-participants  Was anyone else present besides the participants and researchers? | Eight participants requested that their family carer be interviewed at the same time as them therefore eight interviews (one participant involved two family carers) were conducted with patient-carer dyads. | Findings - 5 |
| 16. Description of sample  What are the important characteristics of the sample? e.g. demographic data, date | 21 female; mean age 84 years. The majority of participants were Caucasian (n=26) with one participant being of Afro-Caribbean heritage. | Findings – 5 and table 1 |
| **Data collection** |  |  |
| 17. Interview guide  Were questions, prompts, guides provided by the authors? Was it pilot tested? | Interviews were semi-structured using a topic guide (see supplementary material). Topic guide was piloted with PPI group to check for appropriateness of language. | Methods (data collection) - 4 |
| 18. Repeat interviews  Were repeat interviews carried out? If yes, how many? | No | - |
| 19. Audio/visual recording  Did the research use audio or visual recording to collect the data? | Interviews were audio recorded where consent was given. | Methods (data collection) - 4 |
| 20. Field notes  Were field notes made during and/or after the interview or focus group? | Yes, field notes were made following each interview. | - |
| 21. Duration  What was the duration of the interviews or focus group? | Interviews ranged from 26 minutes to 40 minutes in length. | - |
| 22. Data saturation  Was data saturation discussed? | Yes – data analysis continued until data saturation occurred, with no new ideas or themes emerging. | Methods (data analysis) - 5 |
| 23. Transcripts returned  Were transcripts returned to participants for comment and/or correction? | No | - |
| **Domain 3: analysis and findings** |  |  |
| **Data analysis** |  |  |
| 24. Number of data coders  How many data coders coded the data? | Interviews were coded by JT and managed in groups of seven to make concurrent data collection and analysis manageable. BF independently coded a quarter of all interviews and held frequent discussions with JT to agree thematic development. | Methods (data analysis) - 4 |
| 25. Description of the coding tree  Did authors provide a description of the coding tree? | No but description of framework matrix development given. | Methods (data analysis) - 4 |
| 26. Derivation of themes  Were themes identified in advance or derived from the data? | Themes were derived inductively from the data. | Methods (data analysis) - 4 |
| 27. Software  What software, if applicable, was used to manage the data? | NVivo 11 | Methods (data analysis) - 4 |
| 28. Participant checking  Did participants provide feedback on the findings? | No however findings were discussed with the PPI group to check for resonance. | Methods (Patient and public involvement in this study) - 3 |
| **Reporting** |  |  |
| 29. Quotations presented  Were participant quotations presented to illustrate the themes / findings? Was each quotation identified? e.g. participant number | Yes, pseudonyms used. | Findings – 6 to 12 |
| 30. Data and findings consistent  Was there consistency between the data presented and the findings? | Yes | - |
| 31. Clarity of major themes  Were major themes clearly presented in the findings? | Yes | Findings (framework analysis) – 6 and figure 1 |
| 32. Clarity of minor themes  Is there a description of diverse cases or discussion of minor themes? | All themes discussed in the findings section. | Findings – 6-12 |
